# Long-term outcomes of genicular artery embolization for knee osteoarthritis: 12-month efficacy and secondary outcomes from a randomized sham-controlled clinical trial

**DOI:** 10.1007/s00330-026-12505-8

**Published:** 2026-04-20

**Authors:** Tijmen A. van Zadelhoff, Rianne A. van der Heijden, Sita M. A. Bierma-Zeinstra, Pieter Koen Bos, Edwin H. G. Oei

**Affiliations:** 1 Department of Radiology and Nuclear Medicine, Erasmus MC, University Medical Center Rotterdam, Rotterdam, The Netherlands; 2 Department of General Practice, Erasmus MC, University Medical Center Rotterdam, Rotterdam, The Netherlands; 3 Department of Orthopaedics and Sports Medicine, Erasmus MC, University Medical Center Rotterdam, Rotterdam, The Netherlands

**Keywords:** Embolization, Genicular artery embolization, Knee osteoarthritis, Randomized controlled trial, Synovitis

## Abstract

**Objectives:**

To determine if genicular artery embolization (GAE) is more effective in relieving pain symptoms after 12 months compared to a sham procedure in patients with mild to moderate knee osteoarthritis. A secondary aim was to investigate changes in synovitis observed at 4 months and the correlation with other clinical outcomes.

**Materials and methods:**

A randomized controlled trial included patients with mild to moderate knee osteoarthritis unresponsive to conservative treatment, who were randomly assigned to receive either GAE or sham treatment. Follow-up was at 1, 4, 8, and 12 months. Pain was assessed using the Knee Injury and Osteoarthritis Outcome Score, and synovitis was imaged with contrast-enhanced MRI at baseline, 1, and 4 months. Outcomes were compared using generalized estimating equations.

**Results:**

Fifty-eight patients were included (GAE group: 29, sham group: 29). Pain scores improved from 44.44 (95% CI: 38.87–50.02) at baseline to 65.61 (95% CI: 57.16–74.06) after 12 months in the GAE group and from 42.34 (95% CI: 36.45–48.22) to 58.15 (95% CI: 48.7–67.6) in the sham group. The between-group difference after 12 months was not significant (7.46; 95% CI: −13.63 to −28.56; *p* = 0.25). There were no significant within-group or between-group changes for synovitis.

**Conclusion:**

Patients undergoing GAE and sham GAE both demonstrate an equal sustained pain reduction at 12 months follow-up, with no differences in synovitis reduction or other clinical outcomes. These results suggest a sustained placebo effect in the long term and do not support the clinical implementation of GAE as a treatment for KOA patients.

**Key Points:**

***Question*** *Is GAE more effective than a sham procedure for mild-to-moderate knee osteoarthritis in the long term, and how does it affect synovitis?*

***Findings**** There was no significant difference in pain reduction or synovitis between the two groups*.

***Clinical relevance**** Pain reduction after GAE appears mainly placebo-driven. The placebo effect is sustained up to 12 months. Furthermore, synovitis was unaffected by the procedure. Therefore, our findings do not support clinical implementation*.

**Graphical Abstract:**

**Figure:**
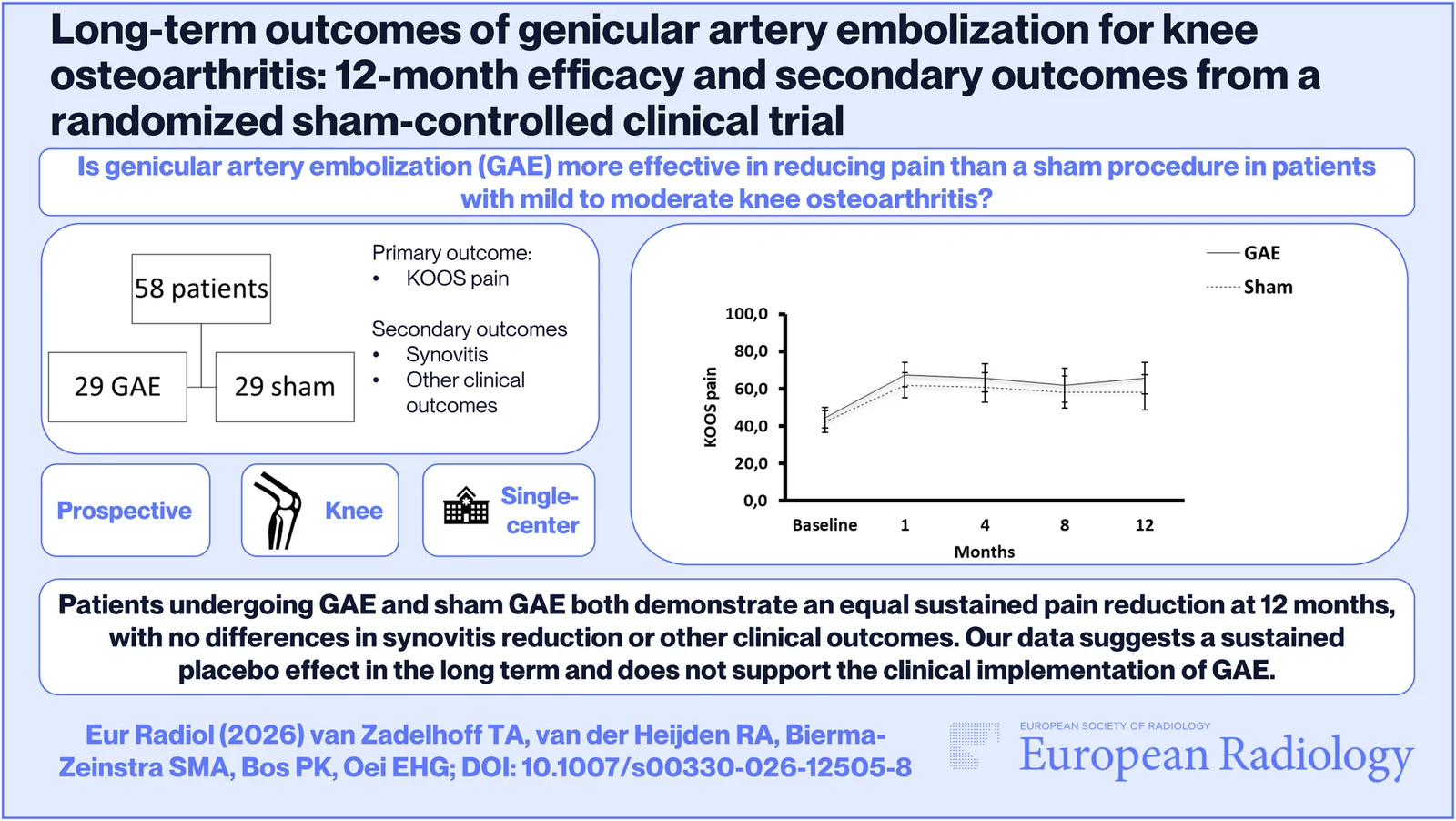

## Introduction

Knee osteoarthritis (KOA) is the most prevalent degenerative joint disorder, resulting in pain, reduced mobility, and diminished quality of life [1]. Conventional treatment options range from conservative approaches such as lifestyle changes, physical therapy, and pharmacological interventions to more invasive procedures, such as knee arthroplasty, in the end stage of the disease [2]. However, there are significant limitations of these treatments, including incomplete pain relief and risks associated with surgery, underscoring the need for alternative therapeutic strategies.

Genicular artery embolization (GAE) is a minimally invasive treatment for KOA that targets peri-genicular hypervascularity, which supplies blood to the synovium, a key contributor to KOA pain [3]. By reducing hypervascularity through endovascular embolization of specific genicular arteries, both synovial inflammation and, as a result, pain are thought to be alleviated [4]. Additionally, perivascular sensory neurons may regress, further reducing pain [3].

Early uncontrolled studies of GAE for KOA showed promising improvements in pain and synovitis, but subsequent sham-controlled randomized clinical trials (RCTs) mostly failed to demonstrate a sustained pain benefit over placebo [5–8]. Overall, the evidence remains inconclusive, with one small trial reporting only a short-term VAS pain advantage at 1 month before sham patients crossed over [9].

We hypothesized that, if there was a true and lasting effect of GAE, the pain reduction in the GAE group would be sustained on longer-term follow-up of our RCT, while the pain in the sham group would relapse. Therefore, the primary objective of this long-term study was to assess whether there was a difference between GAE in reducing pain compared to a sham procedure after 12 months. A secondary aim was to investigate the changes in synovitis observed on magnetic resonance imaging (MRI) and the correlation with other clinical outcomes, providing insight into the working mechanism of GAE.

## Methods

The trial protocol, registered as NCT03884049 (ClinicalTrials.gov), was approved by the Erasmus University Medical Centre ethics committee (MEC identifier: MEC-2018-081). This prospective randomized sham-controlled trial was conducted at Erasmus MC, University Medical Centre Rotterdam, The Netherlands.

### Participants

Patients were recruited at the orthopedic surgery outpatient clinics of Erasmus MC and four regional hospitals (IJsselland Hospital, Maasstad Hospital, Ikazia Hospital, and Spijkenisse Medical Centre). All participants provided written informed consent.

The most important inclusion criteria were: age ≥ 18 years, knee pain for ≥ 6 months, knee pain (NRS ≥ 4 to ≤ 8) on at least half of the days in the preceding month, insufficient response to conservative treatment for ≥ 6 months determined by an orthopedic surgeon and radiographic KOA (Kellgren and Lawrence grade 1–3 [10]).

The most important exclusion criteria were: contraindications for MRI or angiography and previous surgical treatment for KOA (except for knee arthroscopy). The full list of inclusion and exclusion criteria was published previously [11].

### Randomization and blinding

Patients were randomly assigned to GAE treatment or sham treatment, in a 1:1 ratio with random blocks of 4 and 6, using a web-based randomization tool (www.aleaclinical.eu). We did not employ stratified randomization. An email with the allocation was sent to the interventional radiologist performing the procedure. He was the only study team member aware of treatment allocation for the entire study period.

The effectiveness of blinding was assessed using James’ blinding index [12]. Three hours post-intervention, participants indicated their perceived group assignment by selecting one of three options: ‘treatment group’, ‘placebo group’, or ‘I do not know’. Responses were used to compute the blinding index, ranging from 0 (no blinding) to 1 (perfect blinding). Blinding was considered successful if the lower bound of the 95% confidence interval (CI) exceeded 0.5 [12].

### Procedures

**Fig. 1:**
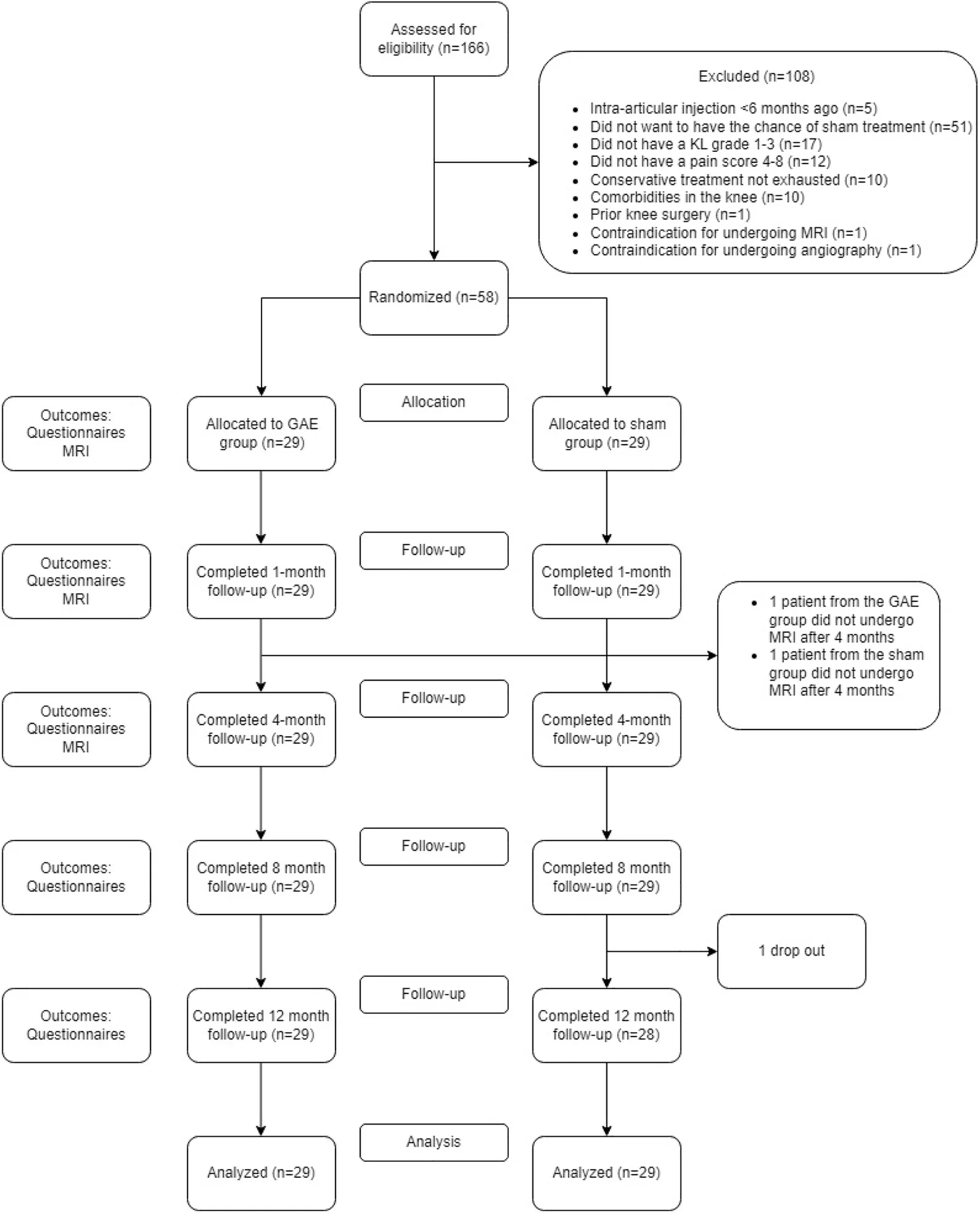
Patient inclusion flow chart

Patients in the intervention group received GAE. A detailed description of the intervention is provided in the published protocol [11]. In short, after local anesthesia, a groin incision was made for vascular access via the common femoral artery. Selective angiography of the genicular arteries was performed using a microcatheter to identify areas of hyperemic blush. Upon visualization, these regions were embolized using 75 or 100 µm Embozene microspheres (Varian). Embolization continued until near-stasis of flow was achieved within the blush, while preserving the surrounding genicular vasculature (Fig. 1). Ice packs were applied to the knee to promote vasoconstriction of small cutaneous arteries and thereby minimize the risk of non-target embolization. All procedures were performed by a single interventional radiologist (A.M.) with 15 years of experience in interventional radiology and embolization. He was not involved in data collection. Sham-group patients underwent a sham GAE procedure. Local anesthesia was administered, and a similar groin incision was made as in the normal procedure. Steps taken during a normal procedure were mimicked, like gaining vascular access, maneuvering the C-arm and table, acquiring images, applying ice packs to the outside of the knee, and preparing and injecting the embolization material. Furthermore, communication between team members was consistent with that in a normal procedure. The sham procedure lasted between 1 h and 2 h, similar to the actual GAE procedure. After the procedure was finished, manual compression was given in the groin area, and a pressure bandage was applied, followed by 3 h rest in the supine position [11].

### Clinical outcomes

Follow-up measurements were collected during in-person visits at Erasmus MC after 1, 4, 8, and 12 months by the same assessor (T.A.v.Z.), blinded to allocation. If patients were unable to attend a follow-up visit, questionnaires were mailed. KOA symptoms were assessed with the validated Dutch Knee Injury and Osteoarthritis Outcome Score (KOOS) questionnaire [13]. The KOOS consists of five subscales: symptoms, pain, daily living, sports and recreational activities, and quality of life. The subscales are summarized in a score ranging from 0 (no complaints) to 100 (worst complaints). Pain, stiffness, and the feeling of knee swelling were evaluated using a Visual Analogue Scale (VAS) ranging from 0 (no pain, stiffness, or swelling) to 100 (worst pain, stiffness, or swelling). To distinguish between intermittent and constant OA pain, the Intermittent and Constant Osteoarthritis Pain (ICOAP) questionnaire was used [14]. The presence of a neuropathic pain component was assessed using the painDETECT questionnaire, with a score of ≤ 12 indicating unlikely neuropathic pain [15]. The EQ-5D-5L questionnaire assessed quality of life [16].

After 12 months, patients were asked to complete the global perceived effect (GPE) questionnaire [17]. This questionnaire is a 7-point Likert scale asking: “To what extent are your complaints changed when compared with the situation just before you started treatment?” Answer options ranged from “worse than ever” to “completely recovered”.

Patients were asked not to start any new therapies during the trial, but doing so would not result in exclusion from the trial. Patients were allowed to stop any ongoing therapies at baseline. Concurrent therapies were documented during all follow-up visits.

### MRI acquisition

MRIs were acquired at baseline, 1, and 4 months with a 3-T MR system (SIGNA Premier, General Electric HealthCare) using a dedicated 18-channel transmit-receiver knee coil (Quality Electrodynamics). Besides morphological pulse sequences to assess structural features of knee OA, a contrast-enhanced (CE) T1-weighted 3D water excitation spoiled gradient-echo sequence (weSPGR) was acquired 6 min after administration of 0.1 mmol/kg gadobutrol (Gadovist, Bayer AG). The scan parameters for this sequence were TR/TE = 10.7/5.4 ms, flip angle = 20°, FOV = 20 × 20 cm, slice thickness = 0.6 mm, and a 512 × 512 mm matrix. Additionally, 2D axial and sagittal fat-suppressed T2-weighted fast spin echo images with an in-plane resolution of 0.4 × 0.6 mm and 3 mm slice thickness were obtained.

### MRI analysis

Synovitis was assessed on CE-MRI using the semiquantitative scoring system of Guermazi et al [18]. Synovial thickness was scored (grade 0: < 2 mm, grade 1: 2–4 mm, and grade 2: > 4 mm) at 11 sites. The sum across all 11 sites yielded the total synovitis score (0–4: normal, 5–8: mild, 9–12: moderate, and ≥ 13: severe). Additionally, effusion synovitis and Hoffa synovitis were scored on the non-contrast fat-suppressed T2-weighted images as described in the MRI Osteoarthritis Knee Score (MOAKS) [19]. Effusion synovitis, defined as hyperintensity within the articular cavity (Grade 0 = none, 1 = small, 2 = medium, and 3 = large), was scored on the axial T2-weighted images. Hoffa synovitis, defined as hyperintensity in Hoffa’s fat pad (0 = normal, 1 = mild, 2 = moderate, and 3 = severe), was scored on the sagittal T2-weighted images. Semiquantitative scoring was performed blinded to group allocation and timepoint. This aimed to minimize any observer bias [20]. All scoring was performed by a single reader (T.A.v.Z.) with 7 years’ experience in musculoskeletal imaging. This reader was trained by a musculoskeletal radiologist (E.H.G.O.) with 20 years’ experience on a separate set of cases. The radiologist was available for consultation on uncertain cases.

### Statistical analysis

An intention-to-treat analysis was conducted. Baseline characteristics were analyzed using descriptive statistics. The primary outcome was the KOOS pain subscale (0-100) after 12 months. To evaluate differences in pain scores between groups over time, a generalized estimating equations (GEE) model was used, accounting for the correlation of repeated measures within individuals. Pain scores at different timepoints were modeled with group, time, and their interaction as predictors. The interaction effect between time point and group determined whether there was a significant difference between groups. Secondary outcomes included synovial enhancement, the other KOOS dimensions (symptoms, daily living, sport and recreation, and quality of life), VAS pain, ICOAP, painDETECT, and the EQ-5D-5L, and were analyzed with the same method. GPE was compared using an independent samples T-test. For comparison of proportions, a chi-squared test was used. The association between synovitis at baseline and a decrease in pain 12 months after treatment in the GAE group was tested using Spearman’s rank. A *p* < 0.05 was considered statistically significant.

## Results

**Fig. 2:**
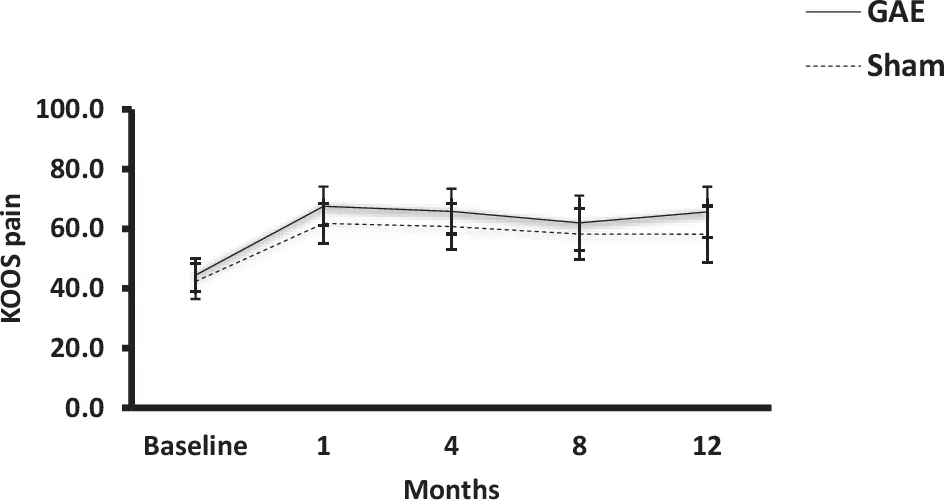
KOOS pain score over time

Details about patient inclusion and drop out are presented in Fig. 2. Between June 2019 and December 2021, 58 patients were included out of 166 screened. One patient dropped out between the 8-month and 12-month follow-up points. Two patients did not undergo MRI at the 4-month follow-up point, but had all other outcomes available. Baseline characteristics are presented in Table 1. James’s blinding index was 0.70 (95% CI: 0.61–1.00), indicating successful blinding.

**Table 1:** Baseline characteristics

		GAE (*n* = 29)	Sham (*n* = 29)
Age		59.01 (8.64)	57.28 (7.95)
Sex	f	19 (65.52)	16 (55.17)
	m	10 (34.5)	13 (44.83)
BMI (kg/m^2^)		29.04 (4.62)	30.99 (4.79)
Laterality	Left	14 (48.28)	16 (55.17)
	Right	15 (51.72)	13 (44.83)
KL grade	1	3 (10.34)	1 (3.45)
	2	9 (31.03)	12 (41.38)
	3	17 (58.62)	16 (55.17)
Duration of complaints in years		7.79 (7.37)	8.17 (8.26)
KOOS pain		44.44 (15.59)	42.34 (16.46)
KOOS symptom		50.25 (17.15)	50.99 (20.95)
KOOS activity		53.1 (15.88)	50.05 (17.98)
KOOS sport		17.68 (16.69)	18.62 (16.79)
KOOS QoL		27.16 (17.38)	27.16 (14.3)
ICOAP constant		44.14 (20.09)	43.45 (20.92)
ICOAP intermittent		49.28 (14.94)	47.84 (20.31)
painDETECT		9.83 (5.22)	9.72 (6.11)
EQ 5D 5 L		0.65 (0.21)	0.61 (0.28)
VAS stiffness		59.48 (24.92)	59.48 (30.95)
VAS swelling		41.21 (29.48)	34.9 (26.66)
Procedure time (min)		107.22 (24.43)	94.39 (16.15)
Contrast agent used (mL)		156.52 (213.45)	-
Radiation (mGy/cm^2^)		6988.59 (7499.71)	-
No. of arteries embolized		3.79 (1.67)	-

Ordinal variables are given as count (%)

*BMI* body mass index, *KL grade* Kellgren and Lawrence grade, *KOOS* knee injury and osteoarthritis outcome score, *ICOAP* intermittent and constant osteoarthritis pain, *VAS* visual analogue scale continuous variables are given as mean (SD)

### Primary outcome

**Fig. 3:**
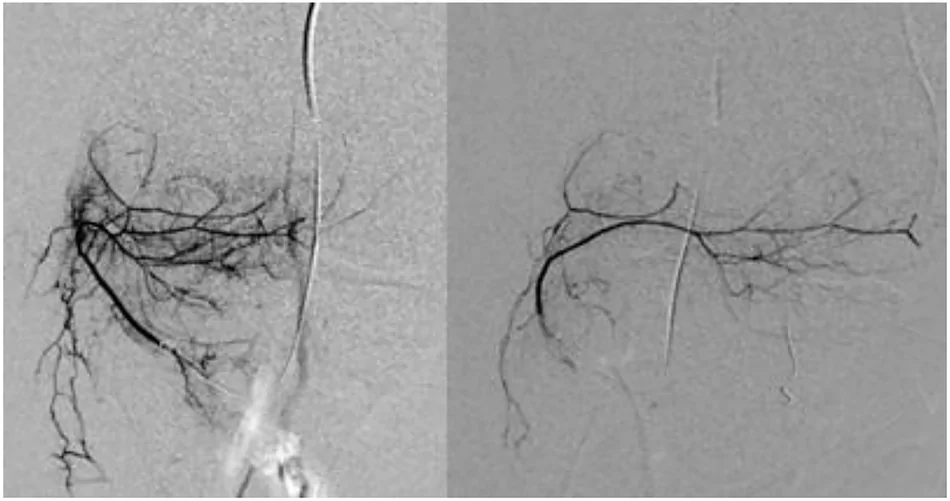
Pre- (left) and post (right) embolization angiography of the lateral inferior genicular artery. Notice the reduction of the hyperemic blush on the postembolization image while the lateral inferior genicular artery was preserved

The primary outcome, KOOS pain at 12 months, did not differ significantly between the GAE and sham groups (between-group difference 7.46 points, 95% CI: –13.63 to 28.56; Table 2). KOOS pain also did not differ between groups at any earlier time point. KOOS pain nevertheless improved substantially in both groups: from 44.44 (95% CI: 38.87–50.02) to 65.61 (95% CI: 57.16–74.06) in the GAE group and from 42.34 (95% CI: 36.45–48.22) to 58.15 (95% CI: 48.70–67.60) in the sham group at 12 months (Fig. 3). At all earlier follow-up visits, both groups also showed significant within-group improvements in KOOS pain compared with baseline (*p* < 0.001 for both groups).

**Table 2:** Comparison of the KOOS score and synovial enhancement between the two groups

		GAE	Sham	Between-group difference	*p*-value
KOOS pain	Baseline	44.44 (38.87–50.02)	42.34 (36.45–48.22)	2.11 (−11.38 to 15.6)	0.61
	1 month	67.53 (60.97–74.09)	61.78 (55.07–68.49)	5.75 (−9.86 to 21.36)	0.23
	4 months	65.8 (58.22–73.39)	60.73 (52.93–68.53)	5.08 (−13.03 to 23.18)	0.36
	8 months	61.97 (52.85–71.1)	58.24 (49.7–66.78)	3.74 (−17.06 to 24.53)	0.56
	12 months	65.61 (57.16–74.06)	58.15 (48.7–67.6)	7.46 (−13.63 to 28.56)	0.25
Synovitis score	Baseline	5.79 (4.45–7.12)	5.45 (4.19–6.7)	0.34 (−2.41 to 3.08)	0.72
	1 month	5.75 (4.21–7.29)	5.48 (4.39–6.58)	0.27 (−2.57 to 3.1)	0.78
	4 months	5.57 (4.04–7.11)	5.89 (4.56–7.22)	−0.32 (−3.36 to 2.72)	0.76

Scores are given as mean (95% CI)

### Secondary outcomes

**Fig. 4:**
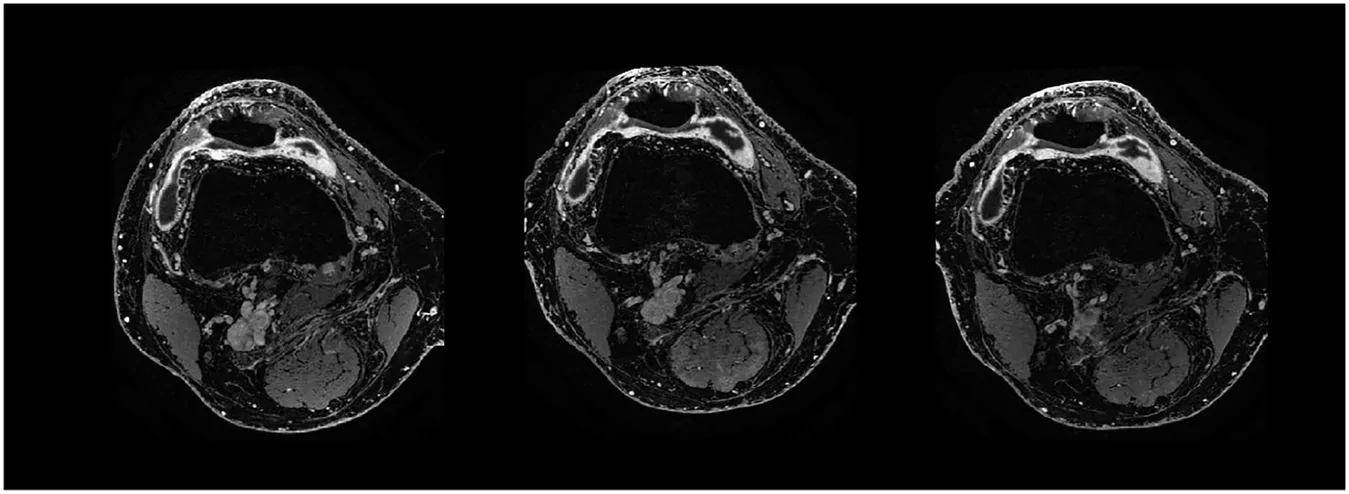
Contrast-enhanced T1 weSPGR images of the knee of a patient from the GAE group at the level of the suprapatellar recess. The baseline image (left) shows a marked synovial enhancement. There was no change 1 month after treatment (middle image) and 4 months after treatment (right image)

Synovitis scores remained essentially unchanged over time in both groups. For the GAE group, mean synovitis score was 5.79 (95% CI: 4.45–7.12) at baseline, 5.75 (95% CI: 4.21–7.29) at 1 month, and 5.57 (95% CI: 4.04–7.11) at 4 months, with no statistically significant within-group change (baseline to 1 month two-sided *p* = 0.94; baseline to 4 months two-sided *p* = 0.59; Fig. 4). In the sham group, synovitis was 5.45 (95% CI: 4.19–6.70), 5.48 (95% CI: 4.39–6.58), and 5.89 (95% CI: 4.56–7.22) at baseline, 1 month, and 4 months, respectively, again without a statistically significant within-group change (baseline to 1 month two-sided *p* = 0.93; baseline to 4 months two-sided *p* = 0.92). No significant between-group differences were observed at any time point (Table 2). There was no significant association between (degree of) synovitis at baseline and decrease of pain 12 months after treatment in the GAE group (ρ: −0.06; *p* = 0.69).

The mean GPE 12 months after treatment for the GAE group (mean 2.79; SD 1.60) and the sham group (mean 3.29; SD 1.61) was not significantly different (*p* = 0.25). There was no statistically beneficial effect of GAE over sham for any KOOS symptom subscale, on intermittent or constant pain, on a neuropathic pain component, or on quality of life. All secondary outcomes are described in Table 3. The usage of co-interventions throughout the study is presented in Table 4. At no point was there a difference in co-intervention usage between the two groups.

**Table 3:** Comparison of questionnaire outcomes between broth groups for the KOOS subscales, a neuropathic pain component (painDETECT), Intermittent and constant pain (ICOAP), quality of life (EQ-5D-5L), and the VAS (Visual analogue scale) for pain

		GAE	Sham	Between-group difference	*p*-value
KOOS symptoms	Baseline	50.25 (44.11–56.38)	50.99 (43.49–58.48)	0.74 (–15.37 to 16.85)	0.88
	1 month	70.44 (64.86–76.03)	67.12 (59.76–74.47)	−3.33 (−18.69 to 12.04)	0.48
	4 months	69.58 (62.77–76.39)	67.98 (60.1–75.86)	−1.6 (−18.93 to 15.73)	0.76
	8 months	67.36 (60.33–74.4)	65.52 (56.9–74.13)	−1.85 (−20.35 to 16.66)	0.74
	12 months	70.32 (63.93–76.71)	67.07 (58.49–75.65)	−3.25 (−21.05 to 14.55)	0.55
KOOS activity	Baseline	52.26 (46.38–58.14)	49.98 (43.33–56.62)	−2.28 (−17.04 to 12.48)	0.61
	1 month	74.95 (68.19–81.71)	69.22 (61.43–77.01)	−5.73 (−22.89 to 11.43)	0.28
	4 months	73.53 (65.38–81.67)	65.58 (57.86–73.29)	−7.95 (−26.61 to 10.72)	0.16
	8 months	70.18 (60.64–79.72)	64.15 (55.95–72.34)	−6.03 (−26.96 to 14.89)	0.35
	12 months	72.01 (63.36–80.65)	65.53 (56.55–74.5)	−6.48 (−27.21 to 14.25)	0.31
KOOS sport	Baseline	16.63 (10.36–22.9)	18.62 (12.62–24.63)	1.99 (−12.45 to 16.43)	0.65
	1 month	35.17 (27.08–43.25)	31.57 (22.12–41.03)	−3.59 (−24.29–17.1)	0.57
	4 months	38.95 (30.23–47.67)	28.87 (19.3–38.44)	−10.09 (−31.63 to 11.46)	0.13
	8 months	33.1 (24.52–41.68)	27.41 (17.85–36.98)	−5.69 (−27.07 to 15.69)	0.39
	12 months	38.37 (26.83–49.9)	33.68 (21.88–45.49)	−4.69 (−32.15 to 22.78)	0.58
KOOS QOL	Baseline	27.16 (20.94–33.37)	27.16 (22.04–32.27)	0 (− 13.39 to 13.39)	1
	1 month	42.03 (35.91–48.14)	39.22 (33.79–44.66)	−2.8 (−16.42 to 10.82)	0.5
	4 months	45.47 (37.97–52.98)	44.83 (37.63–52.02)	−0.65 (−17.95 to 16.65)	0.9
	8 months	46.34 (37.23–55.45)	40.09 (31.44–48.73)	−6.25 (−27.15 to 14.65)	0.33
	12 months	48.28 (39.42–57.13)	40.99 (32.47–49.5)	−7.29 (−27.73 to 13.15)	0.24
painDETECT	Baseline	9.83 (7.96–11.7)	9.72 (7.54–11.91)	−0.1 (−4.88 to 4.68)	0.94
	1 month	8.66 (6.68–10.63)	6.66 (4.83–8.48)	−2 (−6.47 to 2.47)	0.14
	4 months	7.16 (5.1–9.22)	6.07 (4.56–7.57)	−1.09 (−5.34 to 3.16)	0.4
	8 months	8.72 (6.42–11.03)	7 (5.27–8.73)	−1.72 (−6.51 to 3.06)	0.24
	12 months	7.1 (4.72–9.49)	7.03 (5–9.07)	−0.07 (−5.29 to 5.15)	0.97
ICOAP constant	Baseline	44.14 (36.95–51.32)	43.45 (35.97–50.93)	−0.69 (−17.95 to 16.57)	0.9
	1 month	18.45 (11.88–25.02)	25.17 (17.76–32.58)	6.72 (−9.75 to 23.2)	0.18
	4 months	22.07 (13.46–30.68)	25.86 (18.9–32.82)	3.79 (−14.62 to 22.21)	0.5
	8 months	27.76 (18.03–37.49)	28.62 (20.03–37.21)	0.86 (−20.73 to 22.45)	0.9
	12 months	23.97 (15.53–32.4)	28.06 (19.58–36.54)	4.09 (−15.81 to 24)	0.5
ICOAP intermittent	Baseline	49.28 (43.94–54.63)	47.84 (40.58–55.11)	−1.44 (−16.44 to 13.57)	0.75
	1 month	27.44 (20.56–34.32)	30.32 (22.59–38.04)	2.87 (−14.33 to 20.08)	0.59
	4 months	29.6 (21.1–38.1)	31.18 (23.49–38.87)	1.58 (−17.48 to 20.65)	0.79
	8 months	30.46 (21.72–39.2)	35.49 (26.88–44.1)	5.03 (−15.39 to 25.45)	0.42
	12 months	29.6 (21.21–37.98)	31.04 (22.52–39.56)	1.44 (−18.45 to 21.33)	0.81
EQ5D5L	Baseline	0.65 (0.57–0.72)	0.61 (0.51–0.71)	−0.04 (−0.24 to 0.17)	0.56
	1 month	0.8 (0.74–0.86)	0.75 (0.7–0.81)	−0.05 (−0.19 to 0.1)	0.29
	4 months	0.74 (0.64–0.84)	0.73 (0.66–0.8)	−0.01 (−0.21 to 0.19)	0.85
	8 months	0.73 (0.64–0.82)	0.69 (0.61–0.77)	−0.04 (−0.24 to 0.17)	0.55
	12 months	0.69 (0.57–0.81)	0.65 (0.54–0.76)	−0.03 (−0.31 to 0.24)	0.68
VAS pain	Baseline	56.14 (49.9–62.37)	52.69 (45.54–59.84)	−3.45 (−19.22 to 12.33)	0.48
	1 month	33.62 (24.71–42.53)	34.31 (25.21–43.41)	0.69 (−20.5 to 21.88)	0.92
	4 months	33.24 (22.83–43.65)	38.86 (30.15–47.58)	5.62 (−16.96 to 28.21)	0.42
	8 months	37.69 (27.25–48.13)	42.24 (32.2–52.29)	4.55 (−19.55 to 28.65)	0.54
	12 months	34.66 (24.55–44.76)	37.41 (26.82–48.01)	2.76 (−21.61 to 27.12)	0.71

Scores are given as mean (95% CI)

**Table 4:** Cointervention usage

Co intervention	Timepoint	GAE	Sham	*p*-value
Physiotherapy	Baseline	5 (17.2%)	4 (13.8%)	0.72
	1 month	3 (10.3%)	2 (6.9%)	0.64
	4 months	3 (10.3%)	3 (10.3%)	1.00
	8 months	5 (17.2%)	6 (20.7%)	0.74
	12 months	3 (10.3%)	3 (10.3%)	1.00
Brace usage	Baseline	2 (6.9%)	3 (10.3%)	0.64
	1 month	3 (10.3%)	1 (3.4%)	0.30
	4 months	2 (6.9%)	3 (10.3%)	0.64
	8 months	1 (3.4%)	1 (3.4%)	1.00
	12 months	1 (3.4%)	0 (0%)	0.31
Acetaminophen	Baseline	11 (37.9%)	14 (48.3%)	0.43
	1 month	7 (24.1%)	12 (41.4%)	0.16
	4 months	6 (20.7%)	12 (41.4%)	0.09
	8 months	11 (37.9%)	12 (41.4%)	0.79
	12 months	10 (34.5%)	10 (34.5%)	1.00
NSAID oral	Baseline	8 (27.6%)	11 (37.9%)	0.40
	1 month	7 (24.1%)	5 (17.2%)	0.51
	4 months	7 (24.1%)	3 (10.3%)	0.16
	8 months	7 (24.1%)	6 (20.7%)	0.75
	12 months	6 (20.7%)	2 (6.9%)	0.13
NSAID topical	Baseline	2 (6.9%)	1 (3.4%)	0.55
	1 month	1 (3.4%)	0 (0%)	0.31
	4 months	0 (0%)	0 (0%)	-
	8 months	0 (0%)	0 (0%)	-
	12 months	0 (0%)	0 (0%)	-
Opioids	Baseline	0 (0%)	2 (6.9%)	0.15
	1 month	0 (0%)	3 (10.3%)	0.08
	4 months	0 ((0%)	3 (10.3%)	0.08
	8 months	0 ((0%)	1 (3.4%)	0.31
	12 months	1 (3.4%)	2 (6.9%)	0.56

Proportions were compared using a chi-squared test

*NSAID* nonsteroidal anti-inflammatory drug

### Adverse events

Adverse events up to 4 months were reported previously [5]. No additional adverse events were registered in the period between 4- and 12-month follow-up. Two patients in the GAE group reported paresthesia at the lateral ankle after the procedure. For one patient, this resolved spontaneously at the 8-month follow-up time point. For the other patient, this persisted throughout the entire study duration.

## Discussion

In this RCT, we compared pain reduction in patients treated with GAE to sham GAE. After 12 months of follow-up, we found a statistically significant decrease in pain within both groups, but contrary to our hypothesis, no significant difference between the two groups. Furthermore, we found no difference in synovitis scores using CE-MRI between the two groups at 4 months after the treatment. Other secondary outcomes (VAS pain score, ICOAP, painDETECT, EQ-5D-5L, and the GPE) did not show a significant difference either. Our results suggest that the pain reduction observed in patients treated with GAE is mainly due to a placebo effect, and that this placebo effect is sustained after 12 months.

The results of this study, demonstrating that GAE offers no greater clinical benefit than a sham procedure, are of interest to all physicians treating KOA patients. These findings suggest that the therapeutic effects attributed to GAE may be primarily driven by placebo mechanisms rather than by the embolization itself. Remarkably, both groups showed clinically relevant improvement. Although this effect is clearly present in multiple studies without a control group, our study does not provide evidence for an underlying mechanism of action [7, 8, 21, 22].

The placebo effect is a well-documented phenomenon in the treatment of KOA, particularly when assessing pain relief [23]. Interestingly, the invasiveness of a procedure is positively correlated with the magnitude of the placebo effect [24]. More invasive treatments, such as intra-articular injections, tend to elicit stronger placebo responses than less invasive treatments. The relatively invasive nature of GAE could be an explanation for the large placebo effect.

Our findings are similar to a study by Landers et al, also comparing GAE to a sham procedure, in which the median KOOS pain score in the GAE group (*n* = 29) improved from 47.2 at baseline to 66.7 after 12 months, while the sham group (*n* = 30) improved from 47.2 to 61.1 [6]. This difference was not statistically significant, and they concluded that GAE produced no significant benefit over a placebo procedure.

In a recent meta-analysis, the weighted mean difference for VAS pain was −37 (95% CI: −44 to −30) after 1 month and −36 (95% CI: −51 to −22) after 12 months [25]. This is a greater effect than was achieved in the GAE group of our current study: −22.5 after 1 month and −21.5 after 12 months. The possibility exists that in our study, no difference was found between the treatment and control group because the effect achieved in the treatment group was smaller than expected.

We found no significant changes in synovitis scores in patients after GAE. This contrasts with a recent study by Dablan et al, which found a significant decrease in synovial enhancement (from 5.1 to 2.9) on CE-MRI using the same whole-joint semi-quantitative scoring method [26]. Pre-interventional synovial enhancement scores were similar between their study (5.1) and ours (5.8 in the GAE group and 5.5 in the sham group), but post GAE synovial enhancement was markedly different for their study (2.9) compared to ours (5.8). Furthermore, they reported a moderate correlation between pre-procedural synovial enhancement and reduction in pain scores. Patients with more severe pre-procedural synovial enhancement had a greater pain reduction. This correlation is of importance since it could hypothetically be used for patient selection. However, this correlation was not observed in the GAE group of our study. In our study, image analysis was performed blinded for patient information and timepoint (i.e., pre- or postprocedural) to minimize any observer bias. It is unclear whether the image analysis was done blinded to the time point in the study by Dablan et al, and this could be a potential source of bias that explains the discrepant results.

Our results challenge current assumptions about the mechanism of action underlying GAE and raise important questions about its clinical utility. Given the invasive nature, potential risks, and substantial cost of the procedure—estimated at approximately $3095.75 by a US based study and € 3432.37 by a Spanish based study—these results underscore the need for caution before broadly adopting GAE in clinical practice [27, 28] The high cost further amplifies the importance of confirming true therapeutic benefit, especially in resource-constrained healthcare systems. This study highlights the essential role of placebo-intervention controlled trials in validating the efficacy of interventional treatments and supports a more evidence-based, cost-conscious approach to managing KOA.

One potential limitation of the study is the small sample size. While the power calculation – based on the effect of GAE reported in an initial study by Okuno et al—resulted in the required sample size, the treatment group showed a smaller improvement and the control group a larger improvement than anticipated [7]. As a result, it is possible that a smaller effect of GAE may have been obscured due to insufficient statistical power, as the study was not designed to detect such a small effect. A second potential limitation is patient selection based on KL grade rather than specific markers of synovitis, like synovitis on MRI. One could argue that for a procedure to reduce perigenicular synovitis, patient selection should be based on the presence and severity of synovitis. However, since we found no significant correlation between synovitis at baseline and clinical outcome, we strongly believe this is unlikely to have influenced the observed outcomes. Larger, sham-controlled trials with similar methodology are required to establish with greater certainty that there is no difference between GAE and a sham procedure in terms of clinical benefit. In conclusion, GAE lacks long-term efficacy for mild-to-moderate KOA. At 12 months, the GAE and sham GAE groups showed equivalent sustained pain reduction and no difference in objective measures like synovitis or other clinical outcomes. This suggests a robust placebo effect and does not support the clinical implementation of GAE for KOA patients due to a lack of demonstrable benefit over the sham procedure.

## Abbreviations

CI: Confidence interval
GAE: Genicular artery embolization
KOA: Knee osteoarthritis
KOOS: Knee injury and osteoarthritis outcome score
OA: Osteoarthritis
RCT: Randomized clinical trial
VAS: Visual analog scale
